# Efficient Non-Interactive Discrete ReLU over CKKS Using Interpolation Look-Up Table

**DOI:** 10.3390/e28050542

**Published:** 2026-05-11

**Authors:** Zhigang Chen, Xinxia Song, Liqun Chen

**Affiliations:** 1 College of Artificial Intelligence, Ningbo University of Finance and Economics, Ningbo 315175, China; chenzhigang@nbufe.edu.cn; 2 School of Big Data and Software Engineering, Zhejiang Wanli University, Ningbo 315100, China; 3 Department of Computer Science, University of Surrey, Guildford GU2 7XH, UK

**Keywords:** fully homomorphic encryption, CKKS scheme, ReLU function, functional bootstrapping, interpolation-based lookup table

## Abstract

Deploying neural networks on encrypted data requires efficient evaluation of nonlinear activations, especially the ReLU function, without decryption. While the CKKS homomorphic encryption scheme supports packed arithmetic over approximate numbers efficiently, its approximate semantics make direct nonlinear evaluation difficult, and polynomial surrogates often introduce approximation error and non-discrete outputs. In this work, we present a task-specific, non-interactive construction for discrete ReLU evaluation in CKKS by combining modulus-switch-based discretization with interpolation-driven lookup-table (LUT) evaluation. We instantiate this design in two complementary schemes. The first uses trigonometric Hermite interpolation and functional bootstrapping to compute a discrete sign indicator, which is then combined with the encrypted input through conditional multiplication to obtain the ReLU output; this variant is compact and suitable for lightweight settings. The second uses iterative most-significant-bit (MSB) bootstrapping to support larger plaintext moduli and higher-precision regimes through repeated digit extraction. A common enabler of both schemes is a discretization step that maps approximate CKKS plaintexts to a finite integer representation; exactness in our setting therefore refers to exact evaluation over this discretized representation, while the deviation from the original CKKS plaintext is governed by the discretization error analyzed in Lemma 1. Experiments on encrypted MNIST inference and the accompanying LUT/storage analysis indicate that the proposed schemes preserve competitive accuracy relative to polynomial-approximation baselines while maintaining manageable auxiliary storage under the reported parameter settings. These results suggest that interpolation-based discrete activation is a promising alternative to polynomial approximation for selected CKKS-based encrypted inference tasks.

## 1. Introduction

According to their computational granularity, contemporary fully homomorphic encryption (FHE) schemes can be grouped into two classes. The first is word-wise (word-level) FHE, such as BGV [1], BFV [2,3], and CKKS [4]. These schemes excel at efficient word-level linear operations (e.g., matrix multiplication) and use Single Instruction Multiple Data (SIMD) packing [5] to embed many plaintexts into one ciphertext. The second is bit-wise (bit-level) FHE, such as FHEW [6] and TFHE [7], which focus on nonlinear logical operations and support arbitrary functions via Boolean circuit evaluation; each bit is encrypted independently, enabling flexible computation of complex nonlinear functions [8].

By contrast, word-wise FHE encounters substantial difficulties when evaluating nonlinear functions. A common strategy is to approximate these functions with polynomial surrogates, such as the polynomial approximations used in CKKS [9,10,11]. However, such methods are reliable only over narrow domains, such as [−1, 1], and can incur significant errors, especially in the vicinity of 0. Consequently, they are unsuitable for error-sensitive applications such as genomics [12] and finance [13]. Moreover, because CKKS handles nonlinearities via polynomial approximation, it typically incurs additional computational overhead and thus exhibits comparatively lower performance.

The nonlinearity of activation functions constitutes their fundamental rationale and primary design objective, as it enables neural networks to learn and represent complex patterns. Early activation functions, most notably the sigmoid and hyperbolic tangent (tanh) functions, are smooth and everywhere differentiable. These properties are essential for gradient-based backpropagation, and such functions crudely emulate neuronal “on/off” behavior. However, practical use quickly exposed limitations: for inputs of large magnitude, these S-shaped functions saturate, driving gradients toward zero. In deep architectures, such saturation accumulates across layers, impeding parameter updates and producing the well-documented vanishing-gradient problem. The rectified linear unit (ReLU) was subsequently introduced to address these deficiencies.

The ReLU is a pivotal function in machine learning. Building on the scheme of Lee et al., a multiplexed parallel-convolution approach combined with minimax polynomial approximations was implemented to realize an approximate homomorphic ReLU capable of supporting high-precision bootstrapping [14]. This design allows direct reuse of parameters pretrained on plaintext data, obviating any retraining. Furthermore, Lee et al. constructed low-degree minimax polynomials for both ReLU and max-pooling, thereby enabling deep-learning workloads under word-wise fully homomorphic encryption [15].

Beyond using approximation polynomials for nonlinear evaluation, several studies employ polynomial interpolation over the finite field *Z_p_* to enable exact nonlinear computation under BGV/BFV with zero approximation error [16,17]. By contrast, outputs produced by conventional CKKS are typically non-discrete. For example, in homomorphic ReLU, the result should ideally be *x* or 0, yet polynomial approximations often yield intermediate values. This behavior is intrinsic to approximation and is difficult to eliminate, making such methods unsuitable for error-critical applications. Given CKKS’s stronger ciphertext-packing capability relative to BGV/BFV, a natural question arises: can interpolation-based homomorphic evaluation of nonlinear functions be realized within CKKS?

Classical CKKS operates on approximate(non-integer) arithmetic, which precludes interpolation-style computation. Recently, however, “discrete CKKS” has been introduced [18,19], in which computations are performed over discrete data. In certain settings, operating in the discrete domain can be more efficient than operating in the approximate domain because interpolation can be used instead of polynomial approximation. Nonlinear functions are notoriously difficult to evaluate homomorphically due to the absence of low-cost, effective mechanisms, whereas interpolation enables evaluation via lookup tables (LUTs) [20] whose computational cost is largely independent of the target function’s complexity. Consequently, by combining discretization with interpolation, one can efficiently handle discontinuities in target functions.

This paper proposes two non-interactive, strictly discrete ReLU schemes for the CKKS FHE framework, built on interpolation-based lookup tables (LUTs) and bootstrapping. The first scheme employs trigonometric Hermite interpolation to construct a periodic function [21], uses functional bootstrapping to perform an approximate LUT evaluation of the Step function, and combines it with homomorphic multiplication to obtain a strictly discrete ReLU output. It features a simple architecture, linear-size LUTs, and is well-suited to lightweight models. The second scheme leverages an iterative MSB bootstrapping technique to progressively extract the high bits of the plaintext, thereby achieving multi-precision ReLU outputs and accommodating scenarios with stricter accuracy requirements. The paper [21] provides a general trigonometric-Hermite functional bootstrapping framework, while our paper specializes it to a strictly discrete ReLU over CKKS by combining modulus-switch discretization, Step/LUT-based sign extraction, and an iterative MSB variant for multi-precision.

Both schemes admit non-interactive realization, yield exact discrete outputs, and substantially curb ciphertext-noise growth and computational complexity. Experimental results show that, across multiple precision settings, our methods maintain high lookup-table (LUT) accuracy, low computational overhead, and strong inference performance, making them well-suited for deployment in privacy-preserving deep neural networks and underscoring their practical significance.

Our contribution is not a fundamentally new homomorphic primitive, but a task-specific construction for non-interactive discrete ReLU evaluation in CKKS. Building on recent advances in discrete CKKS bootstrapping [19], general functional bootstrapping over CKKS [21], and iterative MSB bootstrapping [22], we specialize these tools to the ReLU setting through a modulus-switch-based discretization step, LUT-based sign extraction, and conditional activation. More specifically, we provide: (i) a functional-bootstrapping-based scheme aimed at compact deployment in lightweight settings, (ii) an iterative MSB-based scheme for larger plaintext moduli and higher-precision regimes, and (iii) an end-to-end evaluation in encrypted neural-network inference. We therefore position the contribution of this paper as a task-specific algorithmic and applied construction rather than as a fundamentally new cryptographic primitive.

## 2. Preliminaries

Let *N* be a power-of-two integer and *Q* > 0 an integer. Define *R* = *Z*[*X*]/(*X^N^* + 1) and *R_Q_* = *Z_Q_*[*X*]/(*X^N^* + 1). We represent *Z_t_* in the centered interval [−*t*/2, *t*/2). In CKKS, a complex message (in *C^N^*^/2^) is first encoded as a plaintext (an element of *R*), and then encrypted as a ciphertext (an element of $R_{Q}^{2}$).

To relate *C^N^*^/2^ and *R*, the CKKS scheme uses the discrete Fourier transform (DFT) and its inverse (iDFT). The $\mathrm{DFT}:R[X]/(X^{N}+1)\to C^{N/2}$ is defined by

$$r(X)\in R[X]/(X^{N}+1)\mapsto {\left(r(\zeta^{i})\right)}_{0\leq i<N/2}\in C^{N/2},$$

where $\zeta_{i}=\zeta^{5^{i}}$ and ζ is a primitive 2*N*-th root of unity. The choice $\zeta_{i}=\zeta^{5^{i}}$ reflects that the automorphism group of the ring *Z*[*X*]/(*X^N^* + 1) is generated by −1 and 5.

The inverse transform (iDFT), i.e., $\mathrm{iDFT}:C^{N/2}\to R[X]/(X^{N}+1)$ is defined as the inverse mapping of the DFT.

The CKKS encoding is defined as follows. Let $z\in C^{N/2}$ be a complex vector. The encoding map $\mathrm{Ecd}:C^{N/2}\to R$ is given by

z↦⌊Δ⋅iDFT(z)⌉∈R,

where $\Delta \in R_{>0}$ is a scaling factor used to preserve precision.

The decoding map $\mathrm{Dcd}:R\to C^{N/2}$ is the approximate inverse of the encoding, defined by

$$m(X)\in R\mapsto \frac{1}{\Delta }\cdot \mathrm{DFT}(m)\in C^{N/2}.$$

This work constructs its schemes using discrete bootstrapping for CKKS. Since discrete bootstrapping need not act on the entire message space over the complex field but only on a prescribed discrete set, one can devise bootstrapping procedures that are more efficient than conventional ones (for example, by using interpolation rather than approximation). We next give a brief overview of the current state-of-the-art implementation of discrete bootstrapping [19].

1. Slots-to-Coefficients (StC). Given a slot-encoded ciphertext ct_slot that encrypts a discrete slot vector *z*, apply the StC transformation to obtain a coefficient-encoded ciphertext ct_coeff_ that encrypts the polynomial whose coefficients represent the entries of *z*. This step changes the representation from slot form to coefficient form.
2. Modulus Raising (ModRaise). Given a coefficient-encoded ciphertext ct under modulus *q*_0_, apply ModRaise to obtain a ciphertext ct_up_ under a larger modulus *Q_top_*. The resulting ciphertext continues to encrypt the same discrete message up to the small perturbation term introduced by modulus raising.
3. Coefficients-to-Slots (CtS). Given a coefficient-encoded ciphertext ct_coeff_, apply CtS to obtain a slot-encoded ciphertext ct_slot_. This step repacks the coefficient information into SIMD slots while adjusting the scaling factor.
4. Homomorphic Exponential (EvalExp). Given a slot-encoded ciphertext ct encrypting a discrete integer message *m*, apply the homomorphic exponential map to obtain a ciphertext ct_exp_ that encrypts $e^{2\pi im_{i}/t}$ in each slot *i*.
5. Homomorphic Look-Up Table (LUT). Given a ciphertext ct and a lookup function f defined on the target discrete domain, apply the interpolation-based LUT procedure to obtain a ciphertext ct*_f_* that encrypts *f*(*m*_i_) in each slot *i*.

For the reader’s convenience, Table A1 summarizes the main notation and parameters used throughout the scheme descriptions, algorithms, and theorem statements.

Security considerations. The proposed discretization, bootstrapping, and LUT-evaluation procedures are public homomorphic transformations applied to ciphertexts under the standard CKKS/RLWE setting. The LUTs used for Step/sign extraction are public functions, and their homomorphic evaluation does not require decrypting intermediate values or introducing data-dependent plaintext control flow. Modulus switching changes the ciphertext modulus and induces the discretized plaintext representation analyzed above, but it does not, by itself, reveal the encrypted value to the evaluator. Similarly, functional bootstrapping and iterative MSB bootstrapping are evaluated using public evaluation keys generated under the usual CKKS key-generation procedure. Therefore, the proposed operations do not change the underlying security assumption of the encryption scheme; the security continues to rely on the hardness assumptions and parameter choices of CKKS. The main additional effect of discretization is numerical rather than cryptographic: it introduces the bounded discretization error discussed in Lemma 1. As in standard homomorphic inference, plaintext information is revealed only to an authorized party that decrypts the final output, while implementation-level side channels are outside the scope of this work.

## 3. Trigonometric-Hermite Interpolation–Based ReLU Scheme Design

Building on the general functional-bootstrapping framework of ref. [21], this section specifies the machinery for a compact discrete ReLU construction over CKKS. This section presents an efficient, non-interactive, and strictly discrete realization of ReLU. The central idea is to apply a modulus-switching–based quantization that maps floating-point inputs to discrete integers, followed by ciphertext-domain sign extraction and table-driven activation, thereby avoiding approximation error and additional multiplicative depth. The scheme is designed for deployment under the CKKS FHE framework. It is particularly well-suited to lightweight inference networks, striking a balance among LUT footprint, ciphertext-precision control, and noise growth, and can serve as a drop-in replacement for ReLU layers, sign layers, or more general conditional activation functions in encrypted inference.

### 3.1. Core Idea and Overall Workflow

Given a CKKS ciphertext encrypting a vector ***z***, we first apply modulus-switch-based discretization to map the approximate plaintext to a finite integer representation. This step converts the input into a form that can be processed by interpolation-based lookup tables while keeping the overall workflow non-interactive. The resulting discretized ciphertext is then passed to a functional-bootstrapping procedure that evaluates a Step function over the discrete domain and returns a binary sign indicator for each slot. Importantly, the binary object produced at this stage is not the ReLU value itself, but an indicator specifying whether the corresponding discretized input is nonnegative.

**Figure 1:**
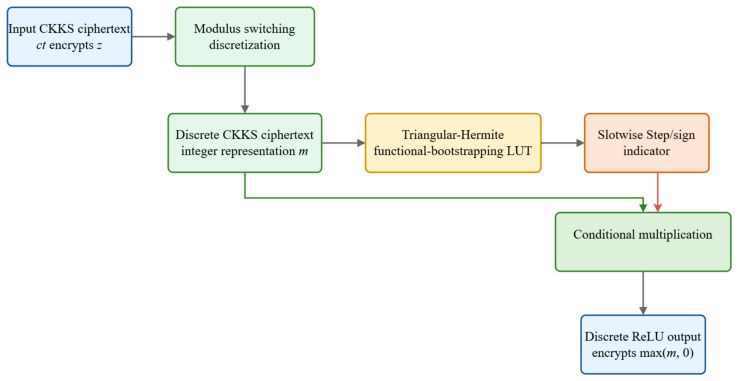
High-level workflow.

The ReLU output is obtained in a final conditional-multiplication step. Once the sign indicator has been computed, we multiply it by the encrypted input, or equivalently by its discretized representative, depending on the implementation level, so that negative slots are mapped to 0, and nonnegative slots retain their corresponding discrete value. Scheme 1 should therefore be understood as a compact three-stage pipeline: discretization, Step/sign evaluation via functional bootstrapping, and conditional activation. Figure 1 summarizes this workflow. Unlike the iterative precision-reduction strategy used in Section 4, the present scheme does not rely on repeated digit extraction and is intended for lower-complexity deployment scenarios.

### 3.2. Modulus Switching and Input Discretization

To enable efficient, non-interactive homomorphic lookups on floating-point inputs (e.g., Step and ReLU activations), we introduce an input-discretization mechanism based on modulus switching. By switching from the current modulus to a smaller one, this mechanism converts a CKKS ciphertext into a discrete-CKKS ciphertext. The ciphertext is then refreshed via a most-significant-bit (MSB) bootstrapping procedure; when functional bootstrapping is employed, it further applies a specified function to the encrypted plaintext.

**Lemma** **1.** *Let ct = (a,b)∈*$R_{q_{0}}^{2}$ *be a CKKS ciphertext (coefficient-encoded) encrypting a vector z∈(−1, 1)^N^ under secret key sk. Set the base scaling factor* Δ_0_ *= q*_0_*. Let q*_1_* be another modulus, and define the discretization procedure* $Discret_{q_{0}}^{q_{1}}=ModSwitch_{q_{1}}^{q_{0}}\circ ModSwitch_{q_{0}}^{q_{1}}:R_{q_{0}}^{2}\to R_{q_{0}}^{2}$ *by*

$$(a,b)\;\to \;\lfloor \frac{q_{0}}{q_{1}}\lfloor \frac{q_{1}}{q_{0}}\cdot (a,b)\rceil \rceil ,$$

*where rounding is coefficientwise. The output* $Discret_{q_{0}}^{q_{1}}$ *encrypts a plaintext m* *∈R*_*q*1_* with m ≈ q·z; the corresponding scaling factor is* $\frac{q_{0}}{q_{1}}$*. In particular,*

$${[<Discret_{q_{0}}^{q_{1}}(\mathrm{ct}),\mathrm{sk}>]}_{q_{0}}={[Discret_{q_{0}}^{q_{1}}(<\mathrm{ct},\mathrm{sk}>)]}_{q_{0}}+e,$$

*where e is the error introduced by discretization and satisfies* ${\left\|e\right\|}_{\infty }=O(\sqrt{h})$ *, with h the Hamming weight of the secret key.*

**Proof.**  Since ct encrypts a vector *z*∈(−1, 1)*^N^* with base scaling Δ_0_ = *q*_0_, multiplying (*a*,*b*) by *q*_1_/*q*_0_ is equivalent to performing one modulus switch, yielding a ciphertext under modulus *q*_1_. Applying a second modulus switch gives $\left\lfloor \frac{q_{0}}{q_{1}}\lfloor \frac{q_{1}}{q_{0}}\cdot (a,b)\rceil \right\rceil$ which corresponds to an encryption (under modulus *q*_0_) of a plaintext *m*∈*R_q_*_1_ that is approximately *m* ≈ $q_{1}\cdot z$. The associated scaling factor is *q*_0_/*q*_1_.

The error introduced by discretizing the ciphertext polynomial is essentially due to rounding, hence each coefficient error is of order *O*(1). When decrypting with a secret key of Hamming weight *h*, the decryption error in a plaintext coefficient can be viewed as the sum of up to *h* independent rounding errors, each of size *O*(1). Consequently, in practice, we obtain the bound ${\left\|e\right\|}_{\infty }=O(\sqrt{h})$. This completes the proof. □

Note that the discretization output is in fact an RLWE ciphertext, which can subsequently be refreshed using the MSB bootstrapping method. In addition, in the sequel, exactness refers to exact evaluation over the discretized plaintext representation unless stated otherwise.

### 3.3. Trigonometric-Hermite Interpolants for the Step and Mod Functions

To implement the conditional logic of ReLU, we must extract the sign of the encrypted input—that is, determine whether the underlying plaintext exceeds zero. To avoid explicit comparison operations, we construct the following Step and Mod functions via trigonometric Hermite interpolation [21].

Construction of the Step function. For *k*∈[*p*]:

$${\mathrm{step}}_{p}(k)=\left\{\begin{array}{ll} 0 & 0\leq k<p/2\\ p/2 & p/2\leq k<p \end{array}\right..$$

Discretized lookup table as below,

$${\mathrm{LUT}}_{\mathrm{step}}=\left[\underset{p/2}{\underset{︸}{0,\ldots ,0}},\underset{p/2}{\underset{︸}{p/2,\ldots ,p/2}}\right].$$

We then employ the trigonometric Hermite interpolation method of Alexandru et al. [21] to construct the periodic interpolant:

$$Rstep_{p}(x)=\frac{p}{4}+\frac{1}{p}\sum_{k\in S}\left(p-k\right)\left(1-i\cot\left(\frac{\pi k}{p}\right)\right)e^{2\pi ikx},$$

where $S=\left\{2i+1:i\in \left[0,\left\lfloor \frac{p}{2}\right\rfloor \right]\right\}$.

Construction of the Mod function. For *k*∈[*p*]:

$${\mathrm{mod}}_{p}(k)={\mathrm{mod}}_{\frac{p}{2}}(k)+{\mathrm{step}}_{p}(k),\;p>2,$$

$${\mathrm{mod}}_{2}(k)={\mathrm{step}}_{2}(k).$$

Discretized lookup tables:

LUTstep*_p_* = [0, …, 0, *p*/2, …, *p*/2] and LUTmod*_p_*_/2_ = [0, 1, …, *p*/2−1, 0, 1, …, *p*/2−1].

Hence, elementwise,

$${\mathrm{LUT}}_{{\mathrm{mod}}_{p}}=\underset{{\mathrm{LUT}}_{{\mathrm{step}}_{p}}}{\underset{︸}{\left[0,\ldots ,0,\;p/2,\ldots ,p/2\right]}}+\underset{{\mathrm{LUT}}_{{\mathrm{mod}}_{p/2}}}{\underset{︸}{\left[0,1,\ldots ,p/2-1,\;0,1,\ldots ,p/2-1\right]}}.$$

### 3.4. Trigonometric-Hermite Interpolation–Based Functional Bootstrapping Algorithm

We adopt the trigonometric-Hermite interpolation–based functional bootstrapping algorithm of Alexandru et al. The input is the ciphertext discretized via modulus switching as described above. The Algorithm 1 proceeds as follows:
**Algorithm 1:** ${\mathrm{FuncBT}}_{q_{0}^{\prime },Q_{L}^{\prime },\Delta }(\mathrm{ct}\in R_{q}^{2},\mathrm{LUT})$Input: ct, a discretized CKKS (or RLWE) ciphertext with ciphertext modulus *q* and plaintext modulus *p*; LUT, the target lookup function on the discrete domain Output: ct′, a bootstrapped ciphertext under modulus Q encrypting the slotwise LUT evaluation1. ct_1_ ← ModSwitch (ct, $q_{0}^{\prime }$) 2. ct_2_ ← $\frac{\Delta }{q_{0}^{\prime }}{\mathrm{ct}}_{1}$ 3. ct_3_ ← ModRaise(ct_2_, $Q_{L}^{\prime }$) 4. ct_4_ ← CtS(ct_3_) 5. ct_5_ ← EvalLUT(ct_4_, LUT) 6. ct_6_ ← StC(ct_5_) 7. ct′ ← ModSwitch(ct_6_, *Q*)

The input ciphertext ct to FuncBT is a discretized CKKS ciphertext (it may also be an RLWE ciphertext) with ciphertext modulus *q* and plaintext modulus *p*. This work chiefly bootstraps discretized CKKS ciphertexts.

To bootstrap, first switch the modulus of ct from *q* to $q_{0}^{\prime }$ (line 1), then adjust the scaling to obtain a CKKS ciphertext whose encoding has the form $\Delta \frac{m(X)}{p}\;\mathrm{mod}\;q_{0}^{\prime }$ (line 2). To allow more homomorphic operations while accommodating noise, raise the ciphertext modulus to $Q_{L}^{\prime }$ (line 3). Next, convert the encoding of ct_3_ from coefficient representation to slot representation (line 4). Then homomorphically evaluate the trigonometric-Hermite interpolation polynomial LUT (line 5). Finally, adjust the ciphertext modulus (lines 6 and 7).

The output ciphertext has ciphertext modulus *Q* and plaintext modulus *P*.

### 3.5. A Multi-Precision Homomorphic ReLU Scheme

In conventional FHE systems, available methods for nonlinear operations support only very low precision. For example, sign-function evaluation based on FHEW/TFHE bootstrapping is typically limited to about 4–5 bits in practice [8]. When higher precision is required, the running time grows roughly linearly with the plaintext/ciphertext modulus (i.e., exponentially in the modulus bit-width). Our ReLU algorithm reduces this dependence to logarithmic complexity, thereby enabling high-precision computation in practice.

Our homomorphic multi-precision ReLU algorithm is as follows. The input ct∈$R_{Q}^{2}$ is a CKKS ciphertext (coefficient-encoded) encrypting a vector *z*∈(−1, 1)*^N^*.

**Theorem** **1.** *Let ct = (a,b)∈*$R_{Q}^{2}$ *be a CKKS ciphertext (coefficient encoding) encrypting a vector z∈(−1, 1)^N^ under secret key sk. In the following theorem, all comparisons involving the encrypted vector z are interpreted slotwise. Let m denote the discretized plaintext representation associated with z after the modulus-switching step of Lemma 1. After applying Algorithm 2, the output ciphertext encrypts the slotwise discrete ReLU of m. More precisely, for each slot j, let z_j_ and m_j_ denote the j-th components of z and of its discretized plaintext representation m, respectively. If z_j_ < 0, the j-th output slot encrypts 0; if z_j_ ≥ 0, the j-th output slot encrypts the corresponding discretized value m_j_ ≈* $P\cdot z_{j}$*. Algorithm 2 invokes FuncBT at most* $\left\lceil \frac{\log P}{\log p}\right\rceil$ *times.*

**Algorithm 2:** HomReLU1(ct∈*R_Q_*^2^) **Input**: ct, a coefficient-encoded CKKS ciphertext encrypting a vector *z* **Output**: ct′, a ciphertext encrypting the slotwise discrete ReLU of the discretized representation of *z*1. ct* ← $Discret_{Q}^{P}(\mathrm{ct})$ 2.   While *Q* > *q* do 3.    ct_1_ ← ct* mod *q* 4.    ct_2_ ← ${\mathrm{FuncBT}}_{q_{0}^{\prime },Q_{L}^{\prime },\Delta }({\mathrm{ct}}_{1},{\mathrm{LUT}(\mathrm{Rmod}}_{p}(x)))$ 5.    ct_3_ ←ct^*^ − ct_2_ 6.    ct* ← ModSwitch(ct_3_, *Q/p*) 7.    *Q* ← *Q/p*, P ← *P/p* 8.  ct_s_ ← ${\mathrm{FuncBT}}_{q_{0}^{\prime },Q_{L}^{\prime },\Delta }(\mathrm{ct}*,\mathrm{LUT}(1-\frac{2}{p}{\mathrm{Rstep}}_{p}(x)))$ 9.  ct′ ← ct_s_ · ct 10.  return ct′

**Proof.**  The encoding of ct* is $\frac{Q}{P}m\;\mathrm{mod}\;Q$, where *m*∈*R_P_* and *m* ≈ P⋅z. After executing line 3 of Algorithm 2, we obtain a ciphertext ct_1_ whose encoding is $\frac{Q}{P}m\;\mathrm{mod}\;q$. Applying the bootstrapping procedure FuncBT (line 4) yields a ciphertext ct_2_ with encoding $\frac{Q}{P}m^{\prime }\;\mathrm{mod}\;Q$, where *m’* = *m* mod *p*. After line 5, we get ct_3_ whose encoding is $\frac{Q}{P}(m-m\;\mathrm{mod}\;p)\;\mathrm{mod}\;Q$. Note that *m*-*m* mod *p* amounts to discarding the lowest log *p* bits of *m* while keeping its higher bits. We then modulus-switch the ciphertext modulus from *Q* to *Q*/*p*, and iterate this loop until the modulus is reduced so that *Q* ≤ *q*. Finally, one more call to FuncBT recovers the most significant bit (the sign) of the plaintext: if z < 0, the output is an encryption of 0; if *z* ≥ 0, the output is an encryption of some *m*∈*R_P_* with *m* ≈ P⋅z. Since each iteration updates *P* ← *P/p*, Algorithm 2 invokes FuncBT at most $\left\lceil \frac{\log P}{\log p}\right\rceil$ times. □

## 4. Design of an Interpolation-Based ReLU Scheme via Iterative MSB Bootstrapping

This section presents a discrete interpolation-based ReLU scheme built on iterative MSB bootstrapping. The core idea is to first use an MSB-bootstrapping primitive to extract the least significant base-*p* digit (with *p* = *t*), and then repeat this procedure *l* times, thereby supporting a plaintext space of size *P* = *t^l^* (the key to multi-precision ReLU). This process enables ciphertext-domain sign extraction, which in turn yields the desired ReLU evaluation.

### 4.1. Core Idea and Overall Workflow

We employ the iterative most-significant-bit (MSB) bootstrapping method of Kim et al. [22], which extends the MSB bootstrapping scheme of Bae et al. [19]. Its chief advantage is that, through iteration, it attains arbitrary-precision bootstrapping—an ability that is crucial for our ReLU framework.

Assume $\mathrm{ct}=(b,a)\in R_{Q}^{2}$ is a coefficient-encoded discrete CKKS ciphertext whose plaintext $m\in R_{t^{l}}$ is embedded in the most significant digits. More precisely, for the secret key sk, we have ${\left[\mathrm{ct}\cdot \mathrm{sk}\right]}_{Q}=\left(\frac{Q}{t^{l}}\right)\cdot m$. We extract the least significant digit iteratively as follows. First, multiply the ciphertext by *t^l^*^−1^ to obtain a CKKS ciphertext whose most significant digits encrypt [*m*]*_t_*. Next, bootstrap this ciphertext (using the integer-to-integer bootstrapping method of [19]) to get an encryption of [*m*]*_t_*. Then apply StC to convert this result to coefficient encoding and subtract it from the original ciphertext ct. At this point, we obtain (i) a slot-encoded CKKS ciphertext encrypting the least significant digit of *m*, and simultaneously (ii) a coefficient-encoded discrete CKKS ciphertext encrypting the remaining high-order part (*m* − [*m*]*_t_*/*t*), which is still positioned in the most significant digits. Repeating this procedure finally yields the sign bit, thereby realizing homomorphic ReLU.

### 4.2. Iterative MSB Bootstrapping Algorithm

We adopt the MSB bootstrapping algorithm of Bae et al. [19]. The input is the ciphertext discretized via modulus switching as described earlier. Algorithm 3 is as follows:
**Algorithm 3:** ${\mathrm{IntBoot}}_{t}(\mathrm{ct}\in R_{q_{0}}^{2},{\mathrm{LUT}}_{\psi^{-1}})$**Input**: ct, a discrete CKKS ciphertext encrypting an integer message over Z_t **Output**: ct′, a bootstrapped ciphertext encrypting the decoded integer or the requested LUT output1. ct_1_ ← ModRaise(ct, $Q_{L}^{\prime }$) 2. ct_2_ ← CtS(ct_1_) 3. ct_3_ ← EvalExp(ct_2_, *ψ* = *x* → *e*^2*πix*/*t*^) 4. ct′ ← LUT(ct_3_, *ψ*^−1^)

The input ciphertext ct to Algorithm 3 is a discrete CKKS ciphertext (coefficient encoding) encrypting a plaintext $m\in R_{t}$, i.e., for the secret key sk, ${\left[\mathrm{ct}\cdot \mathrm{sk}\right]}_{q_{0}}=\left(\frac{q_{0}}{t}\right)\cdot m$. Let *ψ*:*Z_t_* → *C^N^*^/2^ be the mapping defined by *ψ*(*x*) → *e*^2*πix*/*t*^, which is associated with roots-of-unity encoding. Line 1 of Algorithm 3 raises the ciphertext modulus from *q*_0_ to a larger $Q_{L}^{\prime }$ to provide computational headroom for the subsequent bootstrapping. Line 2 converts the ciphertext from coefficient encoding to slot encoding. Line 3 is one of the core steps. It maps the encrypted integer to its corresponding root of unity on the unit circle, enabling the following LUT interpolation to be carried out more stably and compactly on the unit circle. Line 4 decodes the root of unity back to an integer.

### 4.3. Using the Step Function as a Look-Up Table (LUT)

To realize the Step function, simply replace the LUT in line 4 of Algorithm 3, i.e., LUT(ct_3_, *ψ*^−1^), with the LUT for the Step function. Concretely, first map the input message *x* to a root of unity on the complex unit circle:*e*^2*πix*/*t*^ → *x*. Then perform the table lookup that maps this complex root of unity to the Step output:

$$e^{2\pi ix/t}\mapsto \left\{\begin{array}{ll} 0, & 0\leq x<t/2\\ t/2, & t/2\leq x<t \end{array}\right..$$

Chung et al. proposed an efficient LUT evaluation method that represents the table with a low-degree polynomial, thereby mapping roots of unity to integers [20]. Concretely, define an interpolation polynomial $f(u)=\sum_{i=0}^{t-1}a_{i}u^{i}$, where *u* = *e*^2*πix*/*t*^ is the input root of unity and *f*(*u*) = Step(*x*). The coefficients *a_i_* are obtained by interpolation. Specifically, construct the Vandermonde matrix *U* whose entries are powers of a primitive *t*-th root of unity:

$$U=\left[\begin{array}{cccc} 1 & 1 & \ldots & 1\\ 1 & \zeta & \ldots & \zeta^{t-1}\\ \vdots & \vdots & \ddots & \vdots \\ 1 & \zeta^{t-1} & \ldots & \zeta^{\left(t-1)(t-1\right)} \end{array}\right],\;\zeta =e^{2\pi i/t}.$$

Interpolation coefficients can be determined by:

[*a*_0_, *a*_1_, …, *a_t_*_−1_]·*U* = [0, 1, …, *t*−1].

That is, by solving the above linear system, we obtain the polynomial interpolation coefficients *a_i_*. Once the interpolating polynomial is obtained, evaluate *f*(*u*) homomorphically in the ciphertext domain; fast exponentiation techniques (e.g., the Paterson–Stockmeyer method) can be used to reduce the homomorphic multiplicative depth.

### 4.4. Homomorphic ReLU with Arbitrary Precision

Below, we construct a homomorphic multi-precision ReLU algorithm based on the MSB bootstrapping method. The input ct$\in R_{Q}^{2}$ is a coefficient-encoded CKKS ciphertext encrypting a vector *z*∈(−1, 1)*^N^*. Here, *P* = *t^l^* and *p* = *t* denote the plaintext moduli for multi-precision and single-precision, respectively, and their corresponding ciphertext moduli are *Q* and *q*.

The core of Algorithm 4 is a loop over *i* = 0, …, *l* − 1. In each iteration, it invokes IntBoot to extract the least significant base-*t* digit; repeating this *l* times progressively reveals the most significant base-*t* digit. Finally, one additional bootstrapping recovers the sign bit of that most significant digit, thereby completing the ReLU evaluation.
**Algorithm 4:** HomReLU2 (ct∈*R_Q_*^2^)**Input**: ct, a coefficient-encoded CKKS ciphertext encrypting a vector *z*, with plaintext modulus *P* = *t*^*l* **Output**: ct′, a ciphertext encrypting the slotwise discrete ReLU of the discretized representation of *z*1. ct* ← $Discret_{Q}^{t^{l}}(\mathrm{ct})$; 2. *P* = *t^l^*, *p* = *t*; 3. for *i* = 0 to *i* = *l* − 1 4.    ct_1_ ← IntBoot*_t_*(*t^l^*^−1-*i*^ · ct*); 5.    if *i < l* − 1 then 6.     ct* ← ct* − *t^i^*
**·** StC(ct_1_); 7.    end if 8.    ct* ← ModSwitch (ct*, *Q/t*); 9.    *Q* ← *Q/t*, *P* ←*P/t*; 10.   end for 11. ct_s_ ← IntBoot*_t_*(ct*, LUT(1−$\frac{2}{p}{\mathrm{step}}_{p}(x)$); 12. ct′ ← ct_s_ · ct; 13. return ct′

**Theorem** **2.** *Let ct = (a,b)∈*$R_{Q}^{2}$ *be a CKKS ciphertext (coefficient encoding) encrypting a vector z∈(−1, 1)^N^ under secret key sk, and let m denote the discretized plaintext representation associated with z under the parameters of Algorithm 4. As in Theorem 1, all comparisons involving the encrypted vector z are interpreted slotwise. After applying Algorithm 4, the output ciphertext encrypts the slotwise discrete ReLU of m. More precisely, for each slot j, let z_j_ and m_j_ denote the j-th components of z and of its discretized plaintext representation m, respectively. If z_j_ < 0, the j-th output slot encrypts 0; if z_j_ ≥ 0, the j-th output slot encrypts the corresponding discretized value m_j_ ≈* $t^{l}\cdot z_{j}$*. Algorithm 4 invokes IntBoot at most l + 1 times.*

**Figure 2:**
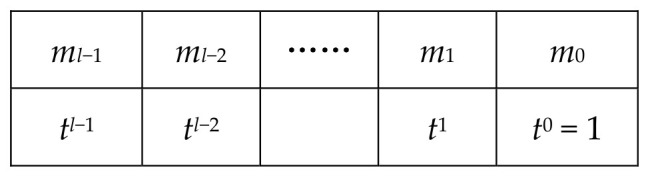
Plaintext form of ct* when *i* = 0.

**Proof.**  Assume $\mathrm{ct}=(b,a)\in R_{Q}^{2}$ is a coefficient-encoded discrete CKKS ciphertext whose plaintext $m\in R_{t^{l}}$ is embedded in the most significant digits. More precisely, for the secret key sk, ${\left[\mathrm{ct}\cdot \mathrm{sk}\right]}_{Q}=\left(\frac{Q}{t^{l}}\right)\cdot m$. We now illustrate one iteration of the loop. When *i* = 0 (before executing line 4), the plaintext form of ct* is as shown in Figure 2.

**Figure 3:**
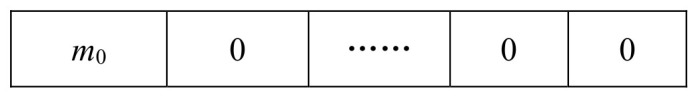
Plaintext shifted right by *l* − 1 digits.

After performing the operation *t^l^*^−1^ · ct*, which corresponds to shifting the plaintext right by *l* − 1 digits, the plaintext takes the form shown in Figure 3.

**Figure 4:**
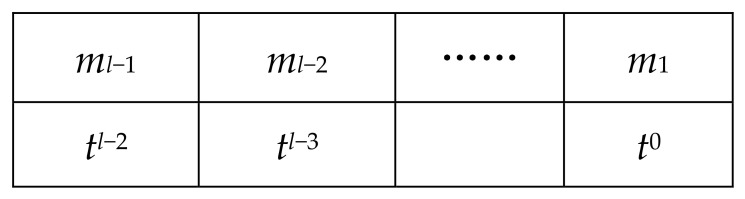
Plaintext form of the new ciphertext ct*.

Thus, we obtain a CKKS ciphertext whose most significant digit encodes *m*_0_ = [*m*]*_t_*. The reason is that ct* encrypts $\frac{Q}{t^{l}}m$; after applying *t^l^*^−1^ · ct*, the underlying plaintext changes from $\frac{Q}{t^{l}}m$ to $\frac{Q}{t}m$, and $\frac{Q}{t}m=\frac{Q}{t}{\left[m\right]}_{t}+\frac{Q}{t}(m-{\left[m\right]}_{t})$. Bootstrapping this ciphertext (line 4) yields a ciphertext ct_1_ encrypting $\frac{Q}{t}{\left[m\right]}_{t}$. Subtracting it from the original ciphertext ct* (line 6) gives a new ciphertext ct* whose plaintext is $\frac{m-{\left[m\right]}_{t}}{t}$, as shown in Figure 4.

Then reduce both the ciphertext modulus and the plaintext modulus (lines 8–9). After exiting the loop, the result is a single-precision ciphertext; one final bootstrapping recovers the sign bit of the most significant digit, thereby completing the ReLU computation. From the number of iterations, Algorithm 4 invokes IntBoot*_t_* at most *l* + 1 times. This completes the proof. □

## 5. Experiments and Discussion

To validate the effectiveness of the proposed interpolation-based, non-interactive, strictly discrete ReLU schemes under fully homomorphic encryption, this section presents a comprehensive experimental evaluation, including detailed setup, performance analysis, and comparative studies. The results demonstrate clear advantages in practical deployment scenarios—most notably, high efficiency and exactness in encrypted model inference.

### 5.1. Experimental Environment and Parameter Settings

All experiments were conducted on a workstation equipped with an Intel Core i9-10900K (3.70 GHz) CPU and 512 GB RAM, running Ubuntu 20.04.3 LTS. The software stack uses the HEAAN fully homomorphic encryption library, with implementations in C++. The specific parameter settings used in our experiments are summarized in Table 1.

In Table 1, N denotes the polynomial ring dimension of CKKS ciphertexts; $h,\tilde{h}$ are the Hamming weights of the dense and sparse secret-key variants, respectively. “CKKS max ciphertext modulus” gives the bit-length of the largest modulus. “Multiplicative depth” indicates the remaining multiplicative depth after bootstrapping (i.e., the number of further multiplications that can still be performed).

### 5.2. Performance Analysis

We profiled the runtime of the two proposed schemes; the measurements are summarized in Table 2.

It is evident that bootstrapping is the primary time consumer in both schemes; however, the trigonometric-Hermite interpolation–based design achieves superior overall runtime compared with the iterative-MSB bootstrapped interpolation ReLU scheme.

### 5.3. Precision and Accuracy Analysis

Because both schemes operate on a discretized CKKS representation, our precision analysis distinguishes the discretization step from the subsequent LUT evaluation. The experiments show that, once the input has been discretized, the proposed procedures produce ReLU outputs that are exact with respect to the resulting discrete representation. Accordingly, when a slot corresponds to a negative discretized input, the output slot is 0; when it corresponds to a nonnegative discretized input, the output slot retains the corresponding discrete value. The remaining deviation from the original approximate CKKS plaintext is inherited from the discretization error analyzed in Lemma 1.

### 5.4. Comparative Experimental Analysis

We further compare the two schemes proposed in this work with mainstream homomorphic ReLU methods; the detailed results are reported in Table 3.

Table 3 compares the proposed schemes with representative CKKS polynomial and TFHE/FHEW-style approaches. This comparison should be interpreted carefully: TFHE-based sign evaluation may achieve lower single-operation wall-clock time, whereas the advantage of the proposed CKKS-based schemes lies in their compatibility with SIMD-packed inference and in the resulting amortized per-slot throughput. Relative to CKKS polynomial approximation, our methods provide exact evaluation over the discretized representation and operate with lower multiplicative-depth overhead for the target activation task. Relative to TFHE/FHEW, the proposed schemes target a different operating point, emphasizing packed CKKS deployment and support for larger precision regimes rather than direct wall-clock superiority for a single operation.

To quantify the auxiliary storage required by the proposed LUT-based activation, Table 4 reports the ReLU-specific LUT/interpolation storage cost under the reported plaintext setting. The table counts only auxiliary lookup values or interpolation coefficients associated with the ReLU evaluation; shared bootstrapping and evaluation keys are not included.

For Scheme 1, the Step and Mod lookup tables contain *p* entries each, so the auxiliary LUT footprint is linear in *p*. With the 8-bit setting *p* = 256, this corresponds to 2*p* = 512 interpolation coefficients, or about 8 KiB if each coefficient is stored as a double-precision complex value. For Scheme 2, the distinct LUTs are determined by the radix *t* and can be reused across iterative rounds; thus, the ReLU-specific storage is linear in t, not in the full plaintext modulus *P* = *t*^l. For example, if *P* = 256 is represented as *t* = 16 and *l* = 2, the distinct LUT footprint is 2*t* = 32 coefficients, or about 512B under the same coefficient-storage convention. Even under a conservative implementation that materializes the digit-decoding LUT separately for each round, the footprint would be at most (*l* + 1)*t* = 48 coefficients, or about 768B.

Reference [14] employs a combination of widely used low-degree minimax polynomials to approximate ReLU. Compared with CKKS polynomial-approximation approaches, our schemes markedly improve computational accuracy, effectively suppress noise growth, and achieve higher efficiency. Relative to TFHE/FHEW methods—although both produce exact outputs—our schemes support higher precision and a larger plaintext space (modulus), while also being more efficient, making them better suited to more complex application scenarios.

Synthesizing the above results, we conclude:

1. Both proposed schemes perform ReLU evaluation exactly with respect to the discretized representation, thereby avoiding the approximation residuals inherent in polynomial-surrogate CKKS methods.
2. Between the two proposed constructions, the trigonometric-Hermite functional-bootstrapping variant offers a more compact activation pipeline, while the iterative MSB variant is better suited to larger precision regimes.
3. Compared with TFHE/FHEW-based sign evaluation, the proposed schemes target a different operating point: they are more naturally integrated with SIMD-packed CKKS inference and can support larger precision settings, but they should not be interpreted as universally superior in single-operation wall-clock runtime.

## 6. Evaluating ReLU Inference in Encrypted Neural Networks

To further validate the applicability and feasibility of the proposed non-interactive, strictly discrete ReLU schemes in practical deep-learning tasks, we adopt the canonical MNIST image-classification dataset and construct a lightweight convolutional network (a LeNet variant). The proposed ReLU schemes are embedded into the homomorphic inference pipeline, and we evaluate inference accuracy, homomorphic computation performance, and scalability under realistic deployment settings.

We use the following three-layer convolutional architecture (simplified LeNet) as the base model:

- Input layer: 28 × 28 grayscale images.
- Convolution 1: 5 × 5 kernels, 8 output channels, ReLU activation.
- Convolution 2: 5 × 5 kernels, 16 output channels, ReLU activation.
- Fully connected: outputs probabilities for 10 classes via softmax.

All ReLU activations are replaced by the proposed non-interactive, strictly discrete ReLU, so they execute directly on CKKS ciphertexts. The ciphertext parameterization is summarized in Table 5.

We evaluated the encrypted inference pipeline on five independent random subsamples of the MNIST test set, each containing 1000 images sampled without replacement within each subsample. For each method, we report the mean and standard deviation of classification accuracy across the five subsamples. The results are summarized in Table 6, where accuracy is reported as mean ± standard deviation.

Intervention F From Table 6, the two proposed ReLU schemes achieve mean encrypted classification accuracies above 96% under the five-subsample evaluation protocol, while maintaining practical inference latency under the reported parameter setting. Compared with the CKKS polynomial-approximation baseline, both schemes show higher mean accuracy and produce exact outputs with respect to the discretized representation. Scheme 1 has lower inference latency, whereas Scheme 2 achieves a slightly higher mean accuracy at the cost of additional computation. The gap to plaintext inference is 2.1 percentage points for Scheme 1 and 1.4 percentage points for Scheme 2, suggesting that the proposed discrete activations preserve competitive inference quality in this experimental setting.

These experiments show that the interpolation-based ReLU is not only theoretically sound but also practically applicable to real neural-network inference. Under ciphertext-level security, it preserves the discreteness and exactness of ReLU while balancing performance and accuracy—appropriate for privacy-preserving image recognition, encrypted medical analytics, and related HE inference scenarios. Our schemes achieve efficiency through three key mechanisms. First, they require significantly fewer bootstrapping operations—Scheme 1 performs only one bootstrap per ReLU activation, and Scheme 2 requires *l* + 1 bootstraps for arbitrary precision, whereas polynomial approximation methods necessitate frequent re-bootstrapping in deep networks to recover computational precision. Second, our LUT-based approach performs function evaluation in a single pass, eliminating the iterative refinement procedures typically required by polynomial methods to achieve acceptable approximation accuracy. Third, discrete ReLU computation consumes minimal multiplicative depth (just one level for the conditional multiplication), avoiding the depth overhead of high-degree polynomial evaluations, which enables the use of larger scaling factors and reduces overall precision loss throughout the network.

Scalability and parameter sensitivity. The dominant cost in both proposed schemes is bootstrapping. In Scheme 1, the main activation cost is one functional-bootstrapping call for Step/sign extraction, followed by one conditional multiplication. Its LUT footprint grows linearly with the single-precision LUT domain size *p*. In Scheme 2, higher precision is supported by representing the plaintext modulus as *P = t^l*; increasing the precision mainly increases the number of iterative bootstrapping rounds, while the distinct LUT footprint is governed by the radix *t*. Thus, Scheme 1 is more compact and suitable for lightweight settings, whereas Scheme 2 trades additional bootstrapping rounds for support of larger plaintext moduli. In both schemes, SIMD packing can improve amortized per-slot throughput, but the wall-clock latency remains dominated by bootstrapping. These trade-offs should be considered when selecting parameters for larger networks or higher-precision inference tasks.

Overall, the results suggest that interpolation-based discrete activation is a promising alternative to polynomial approximation for CKKS-based encrypted inference. The two constructions provide different trade-offs between compactness and precision, and the experiments indicate that both can be integrated into encrypted neural-network pipelines under the reported parameter settings. Future work will focus on broader empirical validation, tighter quantitative analysis of auxiliary storage, and extension of the approach to other nonlinearities and larger architectures.

## 7. Conclusions

This paper presented two non-interactive constructions for discrete ReLU evaluation over CKKS by combining modulus-switch-based discretization with interpolation-based LUT evaluation. The first construction uses trigonometric-Hermite functional bootstrapping to compute a slotwise Step/sign indicator and then obtains the ReLU output through conditional multiplication. The second construction uses iterative MSB bootstrapping to support larger plaintext moduli and higher-precision settings. In both cases, exactness refers to the evaluation over the discretized plaintext representation, while the deviation from the original CKKS plaintext is governed by the discretization step.

The experimental results indicate that the proposed schemes can be integrated into encrypted neural-network inference and can preserve competitive accuracy relative to polynomial-approximation baselines under the reported parameter settings. The two schemes offer different trade-offs: the trigonometric-Hermite variant provides a more compact activation pipeline, whereas the iterative MSB variant is more suitable for higher-precision regimes. Future work will focus on broader empirical validation, tighter storage and runtime analysis, and extensions to other nonlinear activation functions and larger neural-network architectures.

## Figures and Tables

**Table 1:** Specific parameters.

*N*	$(h,\tilde{h}$)	CKKS Max Ciphertext Modulus (Bits)	Multiplicative Depth
2^16^	(192,32)	1280	10

**Table 2:** Runtime (seconds).

Operation	Trigonometric-Hermite Interpolation–Based ReLU Scheme (s)	Iterative-MSB Bootstrapped Interpolation-Based ReLU Scheme (s)
Modulus switching	1	1
Functional bootstrapping	80	87
Conditional multiplication	4	4
Total	85	92

**Table 3:** Runtime and precision comparison of homomorphic ReLU methods.

Method	Runtime (s)	Amortized Time per Slot (ms)	Output Precision
Our Scheme 1 (trigonometric Hermite interpolation)	85	1.3	Exact over discretized representation
Our Scheme 2 (Iterative MSB bootstrapping)	92	1.4	Exact over discretized representation
CKKS polynomial approximation [14]	125	1.91	Approximate
TFHE bootstrapped sign-function scheme [8]	1.4	1400	Exact

**Table 4:** ReLU-specific LUT/interpolation storage cost.

Scheme	Parameters	Distinct LUT/Interpolation Tables	Entries	Estimated Coefficient Storage (Byte)
Scheme 1: trigonometric-Hermite functional bootstrapping	*p* = 256	Step LUT and Mod LUT	2*p* = 512	512 × 16 = 8192
Scheme 2: iterative MSB bootstrapping	*P* = *t*^*l*	digit-decoding LUT and Step LUT, reused across rounds	2*t*	32*t*

**Table 5:** Neural-network ciphertext parameters.

Parameter	Value	Description
Plaintext modulus	256	Supports 8-bit discrete inputs
Ring dimension	2^16^	Supports 32,768-way parallel inference
Bootstrapping	Enabled (per layer)	Restores precision and computational depth

**Table 6:** Encrypted inference on MNIST: performance comparison.

Scheme	Classification Accuracy (Mean ± std, %)	Avg. Inference Time (s)	Activation Precision
Our Scheme 1 (trigonometric Hermite interpolation)	96.2 ± 0.5	16.8	Exact over discretized representation
Our Scheme 2 (Iterative MSB bootstrapping)	96.9 ± 0.4	20.3	Exact over discretized representation
Polynomial approximation (5th degree)	93.5 ± 0.7	26.5	Approximate floating-point output
Plaintext inference (baseline)	98.3 ± 0.3	0.05	Reference for the original network

## Data Availability

Data are contained within the article.

## Appendix A

**Table A1:** Notation table.

Symbol	Meaning
*N*	Ring dimension of the CKKS polynomial ring
*R* = Z[*X*]/(*X*^*N* + 1)	Base polynomial ring
*R_Q_*	Residue ring modulo ciphertext modulus *Q*
*Q*, *q*	Ciphertext moduli used at different levels
*q*_0_, *q*_1_	Moduli used in the modulus-switching discretization step
*Q*_top_	Raised ciphertext modulus used during bootstrapping
*P*	Plaintext modulus for the multi-precision discrete representation
*p*	Plaintext modulus/LUT domain size in the single-precision setting
*t*	Radix used by the iterative MSB bootstrapping scheme
*P* = *t*^*l*	Multi-precision plaintext modulus represented in radix *t*
*l*	Number of iterative rounds in the multi-precision setting
Δ	CKKS scaling factor
*h*	Hamming weight of the secret key
ct, ct′	Input and output ciphertexts
ct_slot_, ct_coeff_	Slot-encoded and coefficient-encoded ciphertexts
z	Slot vector encrypted by the input CKKS ciphertext
*m*	Discretized plaintext/integer representation associated with **z**
sk	Secret key
StC	Slots-to-Coefficients transformation
CtS	Coefficients-to-Slots transformation
ModSwitch	Modulus-switching procedure
ModRaise	Modulus-raising procedure
FuncBT	Functional bootstrapping routine used in Scheme 1
IntBoot	Integer/iterative MSB bootstrapping routine used in Scheme 2
Step	Discrete sign/indicator function used for conditional activation
LUT	Lookup table or its interpolation polynomial
LUT_step_, LUT_mod_	Lookup tables for the Step and modular-reduction related functions
